# Hadronic bound states in SU(2) from Dyson–Schwinger equations

**DOI:** 10.1140/epjc/s10052-015-3324-x

**Published:** 2015-02-28

**Authors:** Milan Vujinovic, Richard Williams

**Affiliations:** 1 Institut für Physik, Karl-Franzens–Universität Graz, Universitätsplatz 5, 8010 Graz, Austria; 2 Institut für Theoretische Physik, Justus-Liebig–Universität Giessen, 35392 Giessen, Germany

## Abstract

By using the Dyson–Schwinger/Bethe–Salpeter formalism in Euclidean spacetime, we calculate the ground state spectrum of $J\le 1$ hadrons in an SU(2) gauge theory with two fundamental fermions. We show that the rainbow-ladder truncation, commonly employed in QCD studies, is unsuitable for a description of an SU(2) theory. This we remedy by truncating at the level of the quark–gluon vertex Dyson–Schwinger equation in a diagrammatic expansion. Results obtained within this novel approach show good agreement with lattice studies. These findings emphasize the need to use techniques more sophisticated than rainbow-ladder when investigating generic strongly interacting gauge theories.

## Introduction

Quantum chromodynamics (QCD) is a strongly interacting gauge theory whose study has proven to be one of the most formidable challenges of modern theoretical physics. While the high-energy regime of QCD is by now relatively well explored in terms of perturbation theory, the arguably more interesting (and intrinsically non-perturbative) phenomena such as dynamical chiral symmetry breaking and confinement are yet to be fully understood.

One of the strategies which might lead to our better understanding of QCD is to investigate theories which are QCD-like, but have certain properties that make them technically less challenging than QCD itself. A prime example is provided by studies of SU(2) gauge theories with an even number of fermion flavors. Lattice simulations of these theories at non-zero chemical potential do not suffer from the sign problem, and such models thus provide ideal conditions to study the phase diagram of strongly interacting matter [1–11].

Here we wish to concentrate on the situation with two fundamentally charged Dirac fermions [1, 8, 10, 11]. Such a theory may also be interesting in the context of a unified description of cold asymmetric Dark Matter (DM) and dynamical electroweak (EW) symmetry breaking [12–14], wherein the ground state hadronic spectrum at $T=0$, $\mu = 0$ is of great importance. It is exactly this hadronic spectrum that will be the central focus of our study.

In this paper we use the non-perturbative, continuous and covariant formalism of Dyson–Schwinger (DSE) and Bethe–Salpeter (BSE) equations in Euclidean spacetime  [15–18]. When applied to QCD, the most common truncation one can make is that of rainbow-ladder (RL), wherein the quark–antiquark interaction kernel is replaced by a dressed one gluon exchange. It is the simplest approximation scheme that respects the axial-vector Ward–Takahashi identity (axWTI), thus preserving the chiral properties of the theory and the (pseudo)-Goldstone boson nature of light pseudoscalar mesons. With a judicious choice of model dressing functions, the RL truncation has been applied relatively successfully to QCD phenomenology for both mesons [19–30] and baryons [31–35].

However, as we will show in this paper, the RL truncation performs unsatisfactorily when adapted to an SU(2) theory with two fundamental flavors, even though the theory is expected to have QCD-like dynamics. We discuss possible reasons for this in more detail in Sect. 2. Here we only comment that we strongly believe that (most) of the inadequacy of RL method comes from its weak connection to the underlying gauge sector. Remedying this requires the use of beyond rainbow-ladder (BRL) techniques, with our preference toward those based on the diagrammatic expansion of quark–gluon vertex DSE [36–46]. While there are other BRL methods available [47–53], we choose the diagrammatic approach as it makes it easier to study the influence of the gauge sector on hadronic observables. Our aim in this paper is thus not only to provide a continuum calculation complementary to the lattice investigations of [12, 13], but also to explicitly demonstrate the importance of using BRL methods when studying generic strongly interacting theories.

This manuscript is organized as follows. In Sect. 2 we discuss the DSEs relevant for our calculation, and also describe in some detail the approximations and model inputs we employ. In Sect. 3 we describe the extrapolation procedures used to obtain hadron masses, and provide estimates for errors coming from extrapolation. The results are discussed and compared to relevant lattice data in Sect. 4. We conclude in Sect. 5.

## Framework

**Fig. 1:**
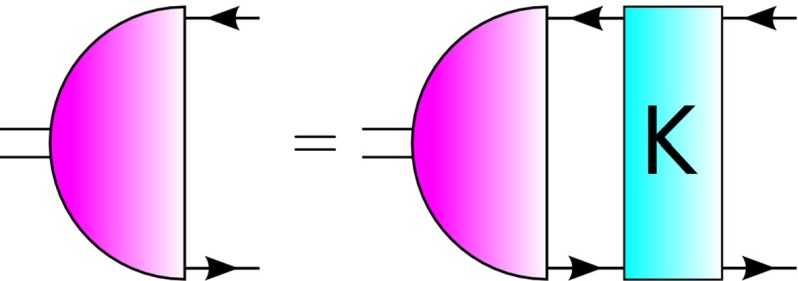
The Bethe–Salepter equation for the meson

In a theory with two colors, both mesons and baryons (diquarks) can be described in terms of a two-body Bethe–Salpeter equation. For the meson

1 $$\begin{aligned} \left[ \Gamma _M(p, P)\right] _{ij} = \int _k\left[ K(p,k,P)\right] _{ik;lj} \left[ \chi _M(k,P)\right] _{kl}, \end{aligned}$$

where $\int _k$ stands for $\int \mathrm{d}^4k/(2\pi )^4$ and $\Gamma _M(p,P)$ is the meson amplitude with appropriate $J^{PC}$ quantum numbers, relative momentum $p$ and total momentum $P$, and the meson wavefunction is $\chi _M(k,P)=S(k_+)\Gamma _M(k,P)S(k_-)$. The quark propagators are $S(k_\pm )$, at momenta $k_+ = k + \eta P$ and $k_- = k - (1-\eta )P$, with $k$ the loop momentum and $\eta \in [0,1]$ the momentum partition factor. In a covariant study, the results are independent of $\eta$: for concreteness, we work with $\eta = 1/2$. The final ingredient in Eq. (1) is the quark–antiquark 4-point interaction kernel $K(p,k,P)$. A diagrammatic representation of Eq. (1) for mesons is given in Fig. 1.

**Fig. 2:**
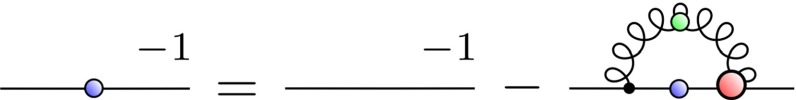
The Dyson–Schwinger equation for the quark propagator. *Straight lines* are quarks, wiggly ones gluons. *Filled circles* indicate dressed propagators and vertices

In order to solve the BSE, one clearly needs as input the quark propagator $S(p)$. This Green function is decomposed as2with $Z_f(p^2)$ the quark wavefunction and $M(p^2)$ the dynamical quark mass. The tree-level form is given by , where $Z_m$ is the quark mass renormalization constant. The quark propagator satisfies its own DSE, see Fig. 2, and is given by

3 $$\begin{aligned}&S^{-1}(p) = Z_2S_0^{-1}(p)\nonumber \\&\quad +\, g^2 Z_{1f}C_F\int _k \gamma ^\mu S(k+p)\Gamma ^\nu (k+p,p)D_{\mu \nu }(k). \end{aligned}$$

Here, $\Gamma ^\nu (p,k)$ and $D_{\mu \nu }(k)$ are the full quark–gluon vertex and gluon propagator, respectively. Renormalization constants of the quark field and quark–gluon vertex are $Z_2$ and $Z_{1f}$. They are related through a Slavnov–Taylor identity which takes a simple form when employing a miniMOM scheme [54] in Landau gauge, $Z_{1f} = Z_2/\widetilde{Z}_3$ with $\widetilde{Z}_3$ the renormalization of the ghost propagator.

The $4$-point interaction kernel $K(p,k,P)$ of Eq. (1) is connected to the self-energy part $\Sigma (p)$ of quark propagator DSE through the axial-vector Ward–Takahashi identity (axWTI)

4 $$\begin{aligned}&[\Sigma (p_+)\gamma _5 + \gamma _5\Sigma (p_-)]_{ij} \nonumber \\&\quad =\int _k \left[ K(p,k,P)\right] _{ik;lj} \left[ \Sigma (k_+)\gamma _5 + \gamma _5\Sigma (k_-)\right] _{kl}. \end{aligned}$$

This identity encodes the chiral properties of the theory, and severely constrains the form of the BSE interaction kernel once an approximation for the quark DSE has been chosen. A direct connection is provided through the action of ‘cutting’ internal quark lines [36, 37].

### Rainbow-ladder

**Fig. 3:**
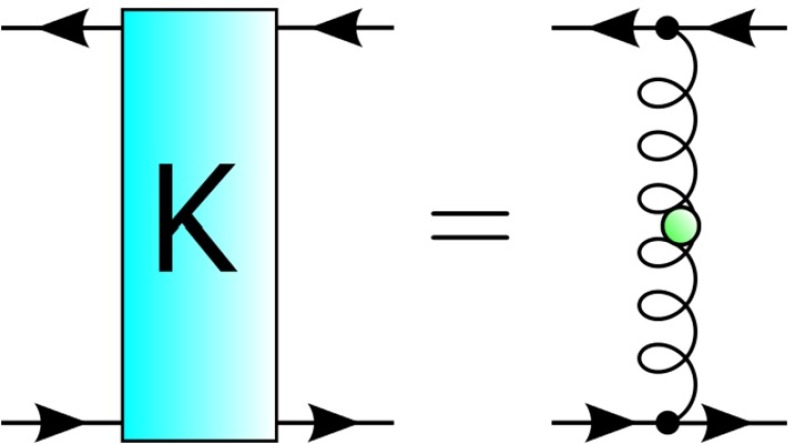
The truncated two-body kernel in rainbow-ladder approximation

The ‘rainbow’ part of RL truncation refers to the replacement of the full quark–gluon vertex in Eq. (3) by

5 $$\begin{aligned} \Gamma ^\nu (k+p,p)\rightarrow \lambda (k^2)\gamma ^\nu , \end{aligned}$$

i.e. its tree-level form augmented by a model dressing function, $\lambda (k^2)$, that is, a function of the gluon momentum alone. The corresponding axWTI-preserving approximation for BSE kernel is that of one gluon exchange (the ‘ladder’), which we show diagrammatically in Fig. 3.

In the RL approach, the model dressing function $\lambda (k^2)$ of Eq. (5) is often combined with the dressing of the gluon propagator $D_{\mu \nu }(k^2)$ into a single model function, constructed to reproduce correctly some hadronic observables, usually $m_\pi$ and $f_\pi$. While this method has shown considerable success in QCD phenomenology (see e.g. [55, 56] for some of the limitations of the model), in an SU(2) theory the approach seems rather unsuitable, especially in the $1^{+\,\!+}$ channel: see Table 2 for details.

There are two primary reasons why RL will not perform satisfactorily in a generic strongly interacting theory. One reason is with regards to its very limited interaction structure ($\gamma ^\nu \times \gamma ^\mu$) which offers no variation in interaction strength across different meson channels. The second is that the connection to the underlying gauge dynamics is typically lost in the construction of an effective quark–gluon interaction; this prevents adequate rescaling of parameters such as $g^2N_c$ that cannot be translated into a re-parameterization of an effective model.

### Beyond rainbow-ladder

**Fig. 4:**
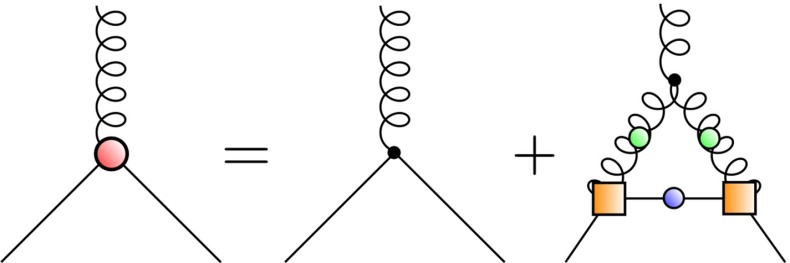
The truncated DSE for the quark–gluon vertex. The *orange square* denotes the internal QG vertex model, according to Eq. (5)

**Fig. 5:**
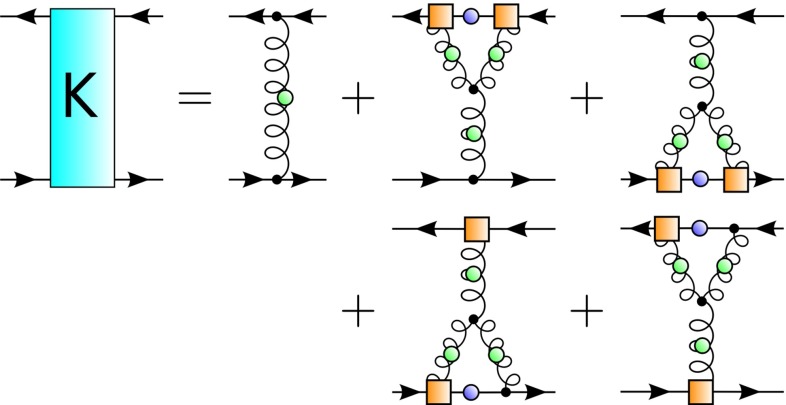
The truncated two-body kernel beyond rainbow-ladder approximation

A BRL approach which is well suited for studying the influence of underlying Yang–Mills sector on the hadronic observables is based on the quark–gluon vertex [41, 44, 46, 57–62]. Here, we focus on the truncated form of the DSE [46] shown in Fig. 4. Within this approximation, only the so-called non-Abelian (NA) diagram is kept in the quark–gluon vertex self-energy. The truncated kernel, consistent with constraints from chiral symmetry, is shown in Fig. 5.

So that the Bethe–Salpeter equation can be tackled, the evaluation of a fully self-consistent quark–gluon vertex is not performed. That is, the full calculated vertex (denoted by a red filled circle in Fig. 4) is not back-coupled into the non-Abelian diagram. Instead, the internal vertices (orange squares in Fig. 4) are modeled by Eq. (5) with $\lambda (k^2)$ constructed such that it strongly resembles the tree-level projection of the full quark–gluon vertex at each iteration step; essentially, it depends upon a function $\Lambda (M_0)$ that encodes the interaction strength in terms of the dynamically generated quark mass. We used the parametrization Eq. (21) of Ref. [46], with modifications that account for the change $N_c=2$ and the rescaling of the gauge coupling $g^2$ ($g^2 N_c$ is left invariant). For $\Lambda (M_0)$ we use the functional form given in Eq. (22) of [46] with parameters $a \simeq 2.44, b \simeq 1.79, c \simeq -0.20, d \simeq 0.30$. The procedure described therein is used for solving the resultant coupled DSE system of a quark propagator and quark–gluon vertex.

We emphasize here that this model is, in a sense, highly constrained. Namely, once the input for the ghost and gluon propagators (which we will discuss shortly) and the truncation of the quark–gluon vertex DSE have been chosen, all other parts of the calculation are fixed. The BSE kernel follows from the axWTI, and the model dressing $\lambda (k^2)$ of Eq. (5) follows from the tree-level projection of the full quark–gluon vertex. We will re-iterate this point in Sect. 4.1, when we provide an estimation of the model dependence.

**Fig. 6:**
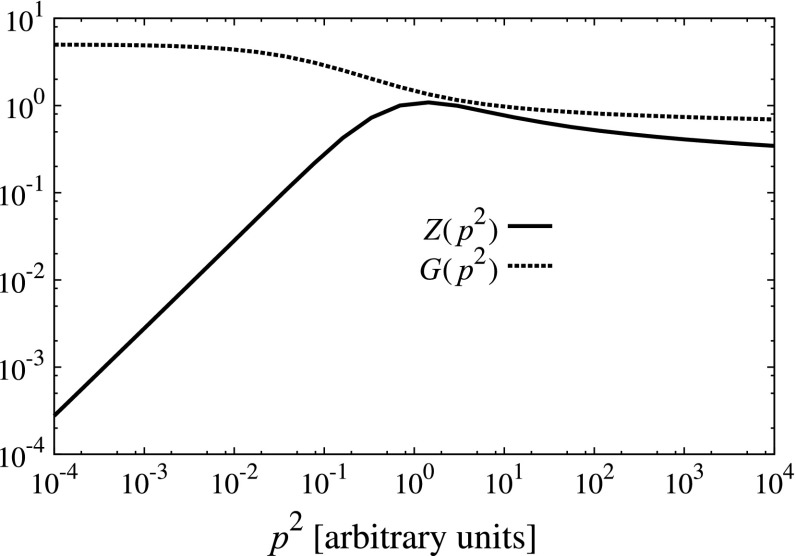
Ghost ($G$) and gluon ($Z$) dressing functions employed in our calculations. The momentum $p^2$ is in arbitrary units: scale setting procedure is described in Sect. 4.2

The final ingredient which we need to specify in our calculation is the gluon propagator $D_{\mu \nu }(k)$. We work in Landau gauge, where this Green function takes the form

6 $$\begin{aligned} D_{\mu \nu }(k) = T_{\mu \nu }(k)\frac{Z(k^2)}{k^2}, \end{aligned}$$

with $T_{\mu \nu }(k) = \delta _{\mu \nu } - k_\mu k_\nu /k^2$ the transverse projector with respect to momentum $k$. The gluon dressing function which we use is plotted in Fig. 6. The details of this function and its parametrization can be found in [63]. We point out that the gluon which we employ corresponds to a quenched DSE calculation. Ignoring the back-reaction of quarks onto the Yang–Mills sector is usually considered a good approximation for theories with QCD-like dynamics, as the corresponding effect on ‘observables’ like the chiral condensate, $f_\pi$ and others is quite small [64]. However, the quenched approximation should be reconsidered in theories which have (nearly) conformal, or ‘walking’ dynamics. Walking dynamics arises naturally in models with a relatively large number of light fundamentally charged fermions [65–73], or fermions belonging to higher-dimensional representations of the gauge group [74–82].

## Bound states from space-like $P^2$

One of the consequences of working with Euclidean spacetime is that access to time-like quantities, such as masses of bound states, requires an analytic continuation of the component Green functions to complex momenta.

While this is only a minor technicality thanks to many well-established techniques in the literature  [22, 49, 83–85], there are situations in which existing methods do not apply, or which are simply too complicated to implement. In this case, indirect methods can be employed that enable access to a limited number of time-like quantities [86, 87].

In the next two sections we describe two techniques that have been widely used, and compare their performance in cases where direct analytic continuation is possible. This provides an estimate of the methods applicability to the study at hand.

### Eigenvalue extrapolation

There are several means by which the mass spectrum of the BSE can be obtained. The most often used is through solution of Eq. (1), written as a matrix equation for simplicity

7 $$\begin{aligned} \Gamma _i = \lambda (P^2) K_{ij}\Gamma _j. \end{aligned}$$

This has solutions at discrete values of the bound state’s total momentum $P^2=-M_i^2$. By introducing the function $\lambda (P^2)$ on the right, we obtain an eigenvalue equation whose bound-state solution correspond to $\lambda (P^2)=1$.

Since $\lambda (P^2)$ is a continuous function of $P^2$, one can conceive that its continuation from space-like $P^2>0$ to time-like $P^2<0$ may be obtained through appropriate function fitting and extrapolation. The transformation of the eigenvalue $g\left( \lambda \right) = 1 - 1/\lambda$, see Ref. [26], removes a considerable degree of intrinsic curvature in the region close to the pole, rendering simple linear extrapolation viable provided the extrapolation is not *far*.

**Fig. 7:**
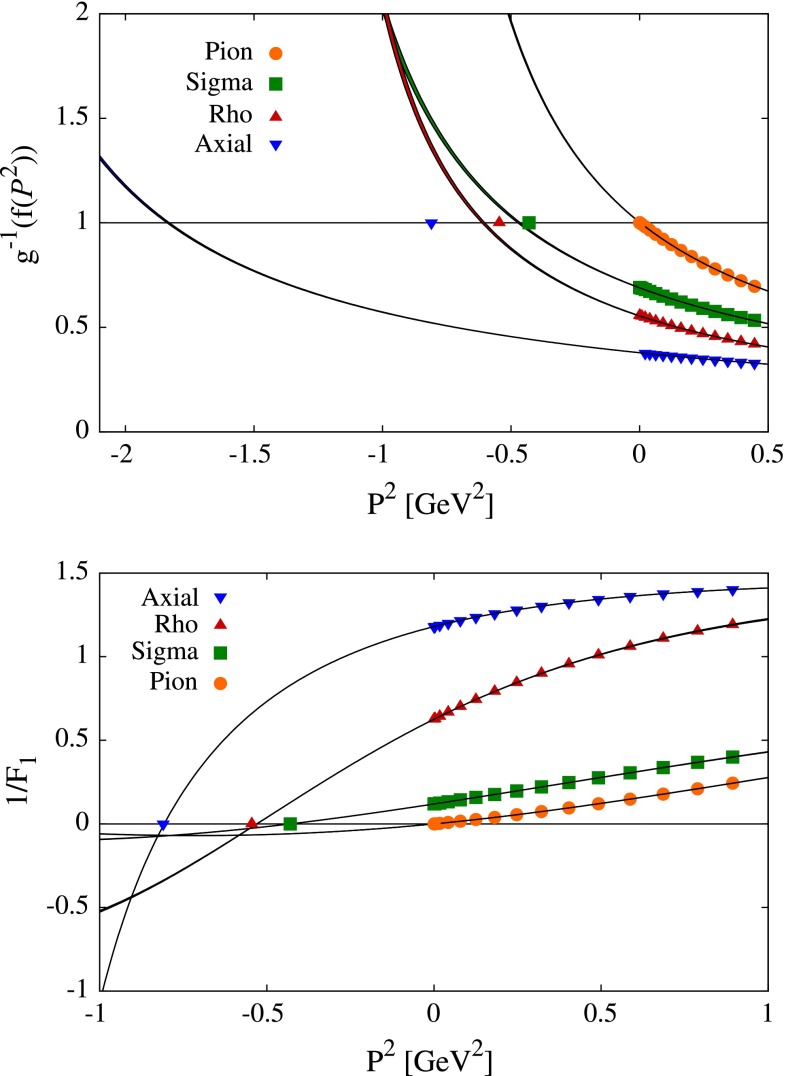
Eigenvalue (*top*) and vertex pole (*bottom*) extrapolation from $P^2>0$ to the time-like region. The known result, obtained by direct analytic continuation, is given by labeled points for comparison

In the top panel of Fig. 7 we show the eigenvalue extrapolation of $\lambda (P^2)$ for various $J^{PC}$ states. The data is first transformed via $g\left( \lambda \right)$, before a linear fit $f(P^2) = a + b x$ is performed. Finally, we plot the inverse function of $g$, $\lambda _{\mathrm {fit}}=g^{-1}(f(P^2))$ as solid lines. Exact results, obtained via calculation in the complex plane, are included as labeled points.

### Inverse vertex function extrapolation

The second means to obtain the mass spectrum employs instead the inhomogeneous BSE for the vertex function $\Gamma _i$

8 $$\begin{aligned} V_i = V^{(0)}_i + K_{ij}V_j. \end{aligned}$$

The obvious difference between this and the homogeneous BSE is the inhomogeneous term $\Gamma ^{(0)}_i$. Its introduction leads to several important changes to the solution. Setting the relative momentum $p$ to zero, for convenience, we observe the appearance of poles

9 $$\begin{aligned} V_i(P^2) \sim \frac{1}{P^2+M^2}, \end{aligned}$$

as one approaches the bound state $P^2\sim -M^2$. Then the determination of a bound-state mass is reduced to looking for zeros in $1/V_i$. Typically, the leading amplitude is used as point of reference, and one employs the method of bi-conjugate gradient (stabilized) for solution.

Restricting ourselves to space-like momenta $P^2$ requires once more the use of fit functions and extrapolation. Here, the most useful are rational polynomials

10 $$\begin{aligned} R^{n,m}(x) = \frac{\sum _{i=0}^n a_i x^i}{1+\sum _{i=1,m}b_i x^i}. \end{aligned}$$

Note, that since the coefficients $a_i$, $b_i$ are obtained through least-squares fitting, the resulting function is not a true Padé approximant. Regardless, the procedure appears quite reliable as can be seen in the bottom panel of Fig. 7.

We summarize our results for vertex pole approximation in Table 1. The results obtained with eigenvalue extrapolation are not quoted as the method performs rather poorly, especially in the $1^{+\,\!+}$ channel (see top panel of Fig. 7). In either of the extrapolation techniques there are two principal sources of uncertainty for the mass values. One comes from the fitting procedure, since the fit function coefficients ($a_i, b_i$ of Eq. (10) for vertex pole method) come with their own error bars. These errors are straightforward to quantify, and the resulting uncertainties for the meson masses are quoted in parentheses in Table 1.

**Table 1:** Results for vertex pole extrapolation for QCD rainbow-ladder in the chiral limit, compared with the result computed through direct analytic continuation. All units are in MeV. The points $P^2$ are taken from the region $(0,L)$; the errors on extrapolated results come from the fitting procedure

$J^{PC}$	Calc	$R^{(2,2)}$ ($L=0.5$)	$R^{(2,2)}$ ($L=1.0$)
$0^{{-}{+}}$	0	$1$	$1$
$0^{{+}{+}}$	658	$657\,(23)$	$656\,(23)$
$1^{{-}{-}}$	738	$731\,(27)$	$728\,(27)$
$1^{{+}{+}}$	900	$899\,(33)$	$899\,(33)$

A second source of errors has to do with the applicability of the extrapolation procedure, as one would expect the whole method to become less reliable as one probes deeper into the $P^2 <0$ region (i.e. the technique is less reliable for heavier mesons). Although it is very hard to quantify this, a comparison with exact results suggests that these effects are quite small for the inverse vertex approximation. In light of other systematic errors, present in both the continuum and lattice investigations of the SU(2) gauge theory, we will ignore this uncertainty in Sect. 4. As an additional check on the extrapolation method, we performed calculations with different fit ranges for $P^2$, with the total momentum sampled in the region $(0,L)$ (in GeV$^2$), and with $L$ given in the table. In the next section we employ the method with $L=0.5$, which appears empirically to have the best performance.

## Results

### Estimation of model dependence

As already highlighted, the majority of model dependence stems from the truncation of the quark–gluon vertex DSE. Other parts of the calculation are constrained either by the underlying gauge dynamics (i.e. the ghost and gluon propagator which are taken from appropriate lattice or continuum calculations) or by chiral symmetry (in the process of truncating the BSE kernel). Thus, we can test the sensitivity of the truncation by varying the solution of the quark–gluon vertex within the constraints imposed by chiral symmetry breaking and the axWTI.

The natural step is to dress the three-gluon vertex. This is motivated by both the 3PI formalism [88] and through the effective resummation of neglected diagrams in the full DSE for the quark–gluon vertex. This in turn enables us to give a rough estimate as to the impact of including additional corrections on our results. It is sufficient to describe the full three-gluon vertex in Landau gauge by its tree structure and one function of a symmetric variable $s_0 = (1/6)\cdot (p_1^2 + p_2^2 + p_3^2)$ [63]

11 $$\begin{aligned} \Gamma ^{\text {3g}}_{\mu \nu \rho }(p_1, p_2, p_3) = \mathcal {A}(s_0)\cdot \Gamma ^{(0)}_{\mu \nu \rho }(p_1, p_2, p_3). \end{aligned}$$

**Fig. 8:**
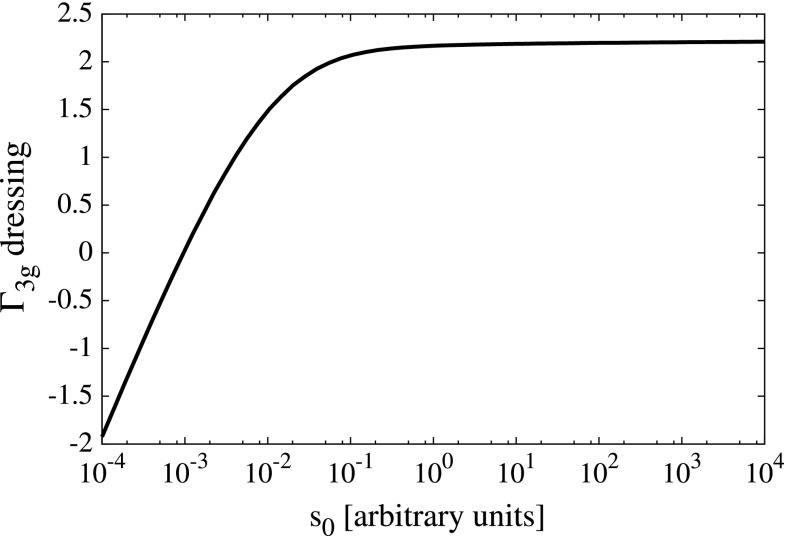
Dressing for the three-gluon vertex, with $s_0 = (1/6)\cdot (p_1^2 + p_2^2 + p_3^2)$ and $a=s=0$; see Eq. (50) of [63]. The momentum variable $s_0$ is in arbitrary units: scale setting procedure is described in Sect. 4.2

**Fig. 9:**
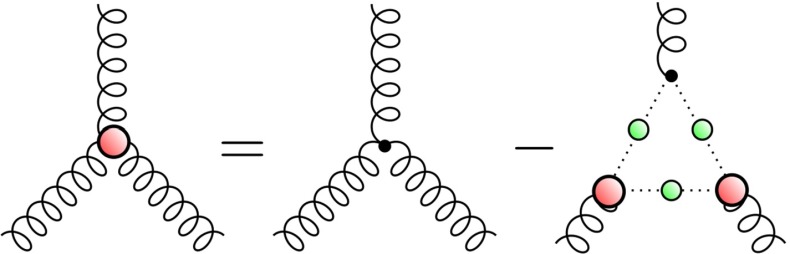
The truncated DSE for the three-gluon vertex. To ensure that bose-symmetry is maintained the right-hand side is averaged over all cyclic permutations

The dressing function $\mathcal {A}(s_0)$ is obtained by solving the three-gluon vertex DSE in a ‘ghost triangle’ approximation, depicted in Fig. 9. The details of the calculation can be found in [63]. The resultant dressing function is shown in Fig. 8. Information available from continuum non-perturbative studies of the three-gluon vertex [63, 89–91] suggests that both the truncation of Fig. 9 and the restriction of possible tensor structures to the tree-level term, provide a reasonable phenomenological description of this Green function. The effect which the dressed three-gluon vertex has on the hadron masses can be seen in Table 2.

There is one further extension to our model that is possible, which is the inclusion of the so-called Abelian diagram in the quark–gluon vertex DSE, see Ref. [46]. This introduces no complications in the evaluation of the quark–gluon vertex itself, and through the ‘cutting’ procedure it is straightforward to construct a solvable BSE kernel which is consistent with axWTI [37]. This BSE kernel would contain diagram with a new topology—the so-called crossed ladder diagram—which increases the algebraic and numerical effort considerably. However, in previous calculations the Abelian contribution has been shown to have a small effect on meson masses, typically less than 2% [92], which would similarly apply to our present investigation. For these reasons, and in light of other uncertainties of continuum and lattice investigations, we feel that it is justified to ignore this extension for now.

**Table 2:** Chiral limit results for meson masses in rainbow-ladder (RL) and beyond rainbow-ladder (BRL) truncations, compared with lattice data for an SU(2) theory. All units are in TeV. Errors of the BRL results come from the extrapolation procedure. For the $0^{+\,\!+}$ state, our continuum result is for an isoscalar; lattice results are forthcoming

$J^{PC}$	RL	BRL, bare 3g vertex	BRL, dressed 3g vertex	Lattice, from [12, 13]
$$0^{{-}{+}}$$	0	$$ 0 $$	$$0 $$	–
$0^{{+}{+}}$	1.24	$1.39\,(6)$	$1.33\,(6)$	–
$1^{{-}{-}}$	1.95	$2.27\,(9)$	$2.36\,(8)$	$2.5 \pm 0.5$
$1^{{+}{+}}$	2.36	$2.87\,(10)$	$3.08\,(10)$	$3.3 \pm 0.7$

### Discussion

Comparison of our results with the lattice [12, 13] requires the scale to be set by equating the electroweak (EW) scale with the pseudoscalar meson (‘pion’) decay constant, i.e. $v_{\mathrm {EW}} = f_{\pi } =246$ GeV. This puts the theory under investigation in the context of dynamical EW symmetry breaking, otherwise known as Technicolor (TC) [93, 94].

The drawbacks that the SU(2) model discussed here (and any other model with QCD-like dynamics) faces as a Technicolor template are by now well known. These include the problems with precision tests on flavor-changing neutral currents [95], and the composite ‘Higgs boson’ which is expected to be very heavy. This latter problem is seen here, whereupon we do not find an isoscalar scalar (‘sigma’) meson (a TC version of the Higgs boson) below $1.33$ TeV. This situation, however, might change drastically if one considers explicitly the couplings to Standard Model particles [96], or more general EW embeddings [97]. Another promising approach to Technicolor phenomenology is to use nearly conformal theories as Technicolor templates [98–101]. As we are presently concerned with the QCD-like aspects of the model under investigation, we will not comment on its possible Technicolor applications further.

Ground state masses for various $J^{PC}$ mesons are shown for both the rainbow-ladder (RL) and beyond rainbow-ladder (BRL) truncations in Table 2, where they are additionally compared with the relevant lattice calculations. RL results were obtained by means of direct analytic continuation, while those of BRL were extrapolated from the region of space-like $P^2$ via the inverse vertex function. The pion decay constant, which is used to set the scale of the calculation, is evaluated via the relation [102]:12where $k_\pm = k \pm P/2$ and $\Gamma _\pi$ is the pion BSE amplitude normalized according to the Nakanishi condition [103]. In QCD, the conventions employed in the above equation would correspond to the value $f_\pi = 93$ MeV. When working in the region of space-like $P^2$, the definition of Eq. (12) can be used without approximations only in the chiral limit, since the pion amplitude $\Gamma _\pi$ can be obtained for the case $P^2 \rightarrow 0$. For non-chiral quarks, and thus non-vanishing pion mass, the calculation would have to be set up for complex total momentum, which is a formidable task in a BRL setting [104].

For the discussion of results it would be useful to have an estimate on the mass of an isoscalar scalar meson, calculated in a method different from our DSE/BSE approach. Since the lattice results for this particle are yet to come, we will use the values obtained by means of group theory scaling, which for the model under investigation gives $m_{\sigma }\in$ $\left[ 1,1.5\right]$ TeV [96]. Taking this into consideration, it seems that the RL method fares well for the sigma meson, and to a lesser extent, the rho meson. In the $1^{+\,\!+}$ channel, this truncation performs inadequately, with a result which deviates by about 30 % from the central lattice value. It is arguable whether or not one can modify the RL method so that it is better suited for an SU(2) theory, thus performing reasonably well for all considered mesons. Based on our current results, and given the limitations of the RL framework, we are skeptical toward this prospect.

On the other hand, the results of the BRL approach compare well with lattice, especially when employing the dressed three-gluon vertex. Since there are considerable error margins present in both the continuum and the lattice investigations, stronger statements about the agreement of our methods will have to wait for more refined calculations.

Regarding the continuum calculation, dressing of the three-gluon vertex seems to lead to a better agreement with the discretized approach, but the overall impact of this modification is relatively mild, and all meson masses are rather robust in this respect. This leaves open the possibility that more elaborate modifications of our model (i.e. inclusion of additional diagrams and higher $n$-point Green functions in the quark–gluon vertex DSE) might not change the results appreciably. However, note that dynamical contributions that can collectively be termed ‘pion cloud’ effects are known to be important, and they are the focus of present and future investigations.

**Fig. 10:**
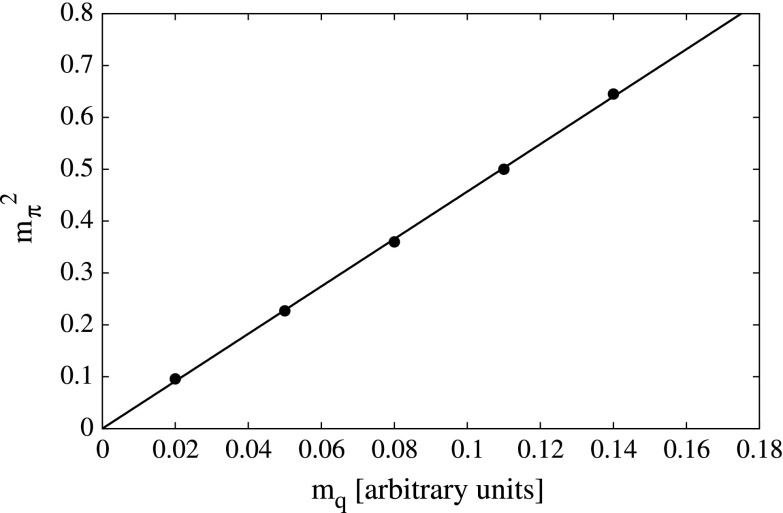
Adherence of the calculated pion mass (*squared*) to the GMOR relation, as a function of the (*corrected*) quark mass $m_q$

**Fig. 11:**
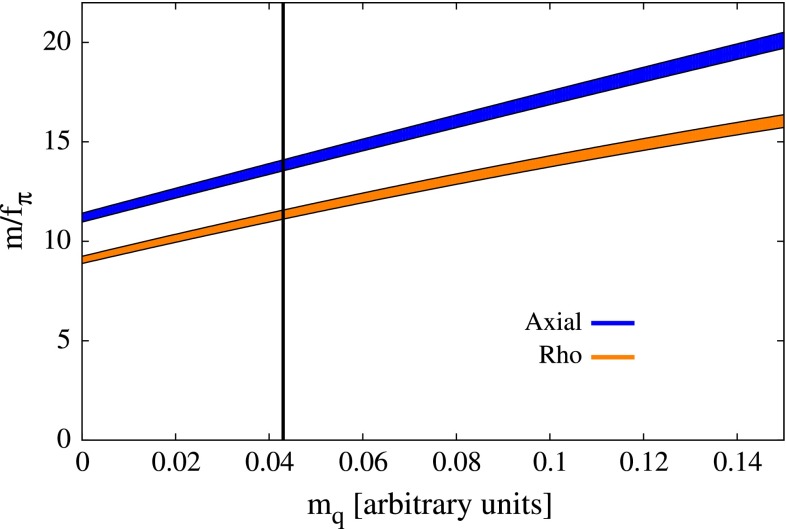
$J=1$ meson masses (in units of chiral limit $f_\pi$) as a function of current quark mass. Bands correspond to uncertainties due to the extrapolation. The right-hand side of the *vertical line* corresponds to the region where $m_\rho \le 2m_\pi$

Apart from the chiral limit study, we also performed calculations for non-vanishing current quark masses. In Fig. 10 we demonstrate the validity of Gell-Mann–Oakes–Renner (GMOR) relation in the BRL approach, while in Fig. 11 we plot the masses of spin one mesons (in units of chiral limit $f_\pi$) as a function of current quark mass. Both plots correspond to a calculation with a bare three-gluon vertex. The results shown in Fig. 11 seem to compare well with the ones shown in Fig. 6 of [13]: however, a direct comparison is not possible since we don’t have enough information to relate our $m_q$ to the ones employed in [13].

As a final remark, we note that the calculation of the baryonic spectrum in this theory does not require any additional effort. An SU(2) gauge theory possesses an enlarged (Pauli–Gürsey) flavor symmetry, which implies that chiral multiplets will contain both mesons and baryons (diquarks). In other words, a meson with $J^P$ quantum numbers will be degenerate with a $J^{-P}$ diquark. This degeneracy (which breaks down if the chemical potential is raised above some critical value $\mu _c$) has been confirmed in numerous lattice investigations [2, 4, 5, 12, 13].

## Conclusions and outlook

We presented a Dyson–Schwinger/Bethe–Salpeter calculation of ground state hadron masses in a theory with two colors and two fundamentally charged Dirac fermions. We employed a novel beyond rainbow-ladder method and obtained good agreement with lattice results for spin one mesons: however, improved calculations will be needed to reduce uncertainties in both lattice and continuum approaches.

For $J=0$ mesons, we demonstrated that chiral dynamics is satisfied (i.e. the GMOR relation holds) and obtained the mass of the sigma meson to be in good agreement with the analysis based on group theory scaling. Additionally, we showed that the rainbow-ladder method performs unsatisfactorily in this strongly interacting template. This underlines the need to use more sophisticated techniques when studying generic non-Abelian gauge theories.

Besides masses, the beyond rainbow-ladder approach we outlined here can also be used to study hadronic decays and form factors. A first step toward accessing these quantities is to extend the calculation to complex Euclidean momenta. However, the technical complications which arise are considerable and are subject to future investigation.
